# Construction of a serum diagnostic signature based on m5C-related miRNAs for cancer detection

**DOI:** 10.3389/fendo.2023.1099703

**Published:** 2023-01-27

**Authors:** Fuzhou Tang, Yang Liu, Yichi Sun, Yu Xiong, Yan Gu, Jing Zhou, Yan Ouyang, Shichao Zhang

**Affiliations:** ^1^ Key Laboratory of Infectious Immune and Antibody Engineering of Guizhou Province, Engineering Research Center of Cellular Immunotherapy of Guizhou Province, Guizhou Medical University, Guiyang, China; ^2^ Immune Cells and Antibody Engineering Research Center of Guizhou Province, Key Laboratory of Biology and Medical Engineering, Guizhou Medical University, Guiyang, China

**Keywords:** liquid biopsy, m5C, serum miRNA, diagnosis, pan-cancer

## Abstract

Currently, no clinically relevant non-invasive biomarkers are available for screening of multiple cancer types. In this study, we developed a serum diagnostic signature based on 5-methylcytosine (m5C)-related miRNAs (m5C-miRNAs) for multiple-cancer detection. Serum miRNA expression data and the corresponding clinical information of patients were collected from the Gene Expression Omnibus database. Serum samples were then randomly assigned to the training or validation cohort at a 1:1 ratio. Using the identified m5C-miRNAs, an m5C-miRNA signature for cancer detection was established using a support vector machine algorithm. The constructed m5C-miRNA signature displayed excellent accuracy, and its areas under the curve were 0.977, 0.934, and 0.965 in the training cohort, validation cohort, and combined training and validation cohort, respectively. Moreover, the diagnostic capability of the m5C-miRNA signature was unaffected by patient age or sex or the presence of noncancerous disease. The m5C-miRNA signature also displayed satisfactory performance for distinguishing tumor types. Importantly, in the detection of early-stage cancers, the diagnostic performance of the m5C-miRNA signature was obviously superior to that of conventional tumor biomarkers. In summary, this work revealed the value of serum m5C-miRNAs in cancer detection and provided a new strategy for developing non-invasive and cost effective tools for large-scale cancer screening.

## Introduction

Most cases of cancer are initially diagnosed at an advanced stage, thus missing the optimal opportunity for therapy and resulting in a dismal prognosis (1). Great efforts have been made in the identification of early diagnostic markers to decrease cancer-specific mortality rates, prolong the survival of patients with cancer, and reduce the societal burden (2). However, the current methods for cancer screening have several disadvantages, such as high costs, poor patient compliance, strong invasiveness, and low accuracy, which limit their feasibility for clinical use in mass cancer screening (3). Given that early diagnosis is a key factor for reducing cancer-related mortality, there is an urgent need to identify a novel biomarker with greater validity and lower invasiveness for large-scale cancer screening.

RNA modifications, which are mediated by different types of regulators (“writers,” “readers,” and “erasers”), carry significant gene regulation-related information, and they play critical roles in tumor occurrence and progression and immune dysregulation (4). 5-Methylcytosine (m5C) modification, an important type of RNA modification, is widely detected in messenger RNAs, transfer RNAs, ribosomal RNAs, long non-coding RNAs, small nuclear RNAs, and microRNAs (miRNAs) (5). The dysregulation of m5C levels was reported to be associated with tumorigenesis and tumor progression. Sun et al. reveled that NSUN2, an m5C methyltransferase, is significantly upregulated in hepatocellular carcinoma (HCC), and it promotes tumor progression by catalyzing the H19 lncRNA methylation-mediated recruitment of the G3BP1 oncoprotein (6). Chen et al. indicated that m5C drives the pathogenesis of bladder urothelial carcinoma (BLCA) by inducing oncogene (e.g., HDGF) activation (7). Moreover, m5C levels can characterize the immune microenvironment infiltration patterns of multiple tumors, and m5C regulators can serve as prognostic and diagnostic markers of cancer (8–10). Recent studies illustrated that m5C acts as a key posttranscriptional modification that facilitates the processing of primary miRNAs. miRNA dysregulation induced by m5C is closely associated with many pathological processes that result in cancer. Zhuo et al. found that m5C could induce an interaction between miR-200c-3p and Argonaute protein, affecting the development of pancreatic cancer (11). Cheray et al. discovered that miR-181a-5p loses its tumor suppressor function upon cytosine methylation in glioblastoma multiforme (12). Liu et al. suggested that zinc-finger E-box binding homeobox 1, an m5C “reader,” promotes tumor progression by suppressing miR-205/miR-200b maturation in HCC (13). Considering the high stability of circulating miRNAs in serum, the identification of novel diagnostic biomarkers for mass tumor screening based on m5C-related miRNAs (m5C-miRNAs) is a promising strategy.

In this study, we used 16,902 serum samples containing 12 tumor types (n = 6607) and 10295 non-tumor serum controls to construct an m5C-miRNA signature for cancer detection. The m5C-miRNA signature both displayed high diagnostic power and presented excellent accuracy in distinguishing tumor types. More importantly, in the detection of early-stage cancers, the diagnostic performance of the m5C-miRNA signature was obviously superior to that of conventional tumor biomarkers. This work revealed the value of serum m5C-miRNAs in tumor detection, and a novel biomarker with excellent accuracy, great effectiveness, less invasiveness, and low cost for large-scale cancer screening was constructed.

## Methods

### Data acquisition and pre-processing

Serum miRNA expression data and the corresponding clinical information (such as age, sex, and TNM stage) were acquired from the Gene Expression Omnibus (GEO) repository (GSE164174, GSE113740, GSE137140, GSE139031, GSE122497, GSE112264, GSE113486, GSE106817, GSE124158, and GSE73220). To eliminate the differences between different platforms, all serum samples were based on the 3D-Gene Human miRNA V21_1.0.0 (GEO accession number GPL21263) platform. We excluded data for patients who received any prior anticancer treatment (such as chemotherapy, radiotherapy, and surgery). The “ComBat” algorithm of the sva package was adopted to remove batch effects (14). In total, 16,902 serum samples covering 12 tumor types [gastric cancer (GC), HCC, lung cancer (LC), glioma, esophageal carcinoma (ESCA), prostate adenocarcinoma (PRAD), BLCA, ovarian cancer (OV), sarcoma (SARC), invasive breast carcinoma (BRCA), colorectal cancer (CRC), and pancreatic adenocarcinoma (PAAD); n = 6607] and non-tumor controls (n = 10,295) were collected.

### Gene ontology enrichment analyses of target m5C-miRNAs

Based on previously published studies, 104 m5C-miRNAs were extracted for subsequent analysis (15–18). GO functional annotation was conducted using the clusterProfiler R package to explore the biological processes and biological functions related to these m5C-miRNAs. FunRich 3.1.1 software was used to identify miRNAs, and then GO enrichment analysis was conducted by utilizing the predicted target genes (19). Remarkably enriched GO terms were determined on the basis of false-discovery rate (FDR) < 0.05.

### Establishment of a diagnostic signature for large-scale cancer screening

To construct a serum diagnostic signature for large-scale cancer detection, 16902 serum samples from microarray data were first randomly assigned to the training or validation cohort at a 1:1 ratio. Randomization was performed using the createDataPartition function of caret R package. The training cohort included 3318 tumor samples and 5173 non-tumor samples, whereas the testing cohort contained 3289 tumor samples and 5122 non-tumor samples. Moreover, we constructed an external validation cohort consisting of 268 tumor samples and 410 non-tumor samples from the combined training and testing cohort. In the training cohort, we used the limma package to evaluate differences in the expression of m5C-miRNAs between tumor and non-tumor samples. Next, differentially expressed m5C-miRNAs (*p* < 0.05 and |fold change| > 1) were selected for further analysis. Finally, 14 candidate m5C-miRNAs were identified by least absolute shrinkage and selection operator (LASSO) regression analyses, and then the diagnostic signature was established using a support vector machine (SVM) algorithm based on these miRNAs (20, 21). The codes for the final model were shown in Supplementary Code. The SVM algorithm was used to classify binary samples (tumor samples vs. non-tumor samples) *via* the “kernlab” R package in R software. Briefly, according to the SVM algorithm, the high-dimensional spatial locations of all samples were determined, for which the expression of each miRNA was indicated by its location on the axis. During training, a hyperplane that best distinguished the two classes was drawn on the basis on the distance between the hyperplane and the nearest sample for each class. Samples from different classes were on different sides of this hyperplane. The diagnostic performance of the m5C-miRNA signature was determined by the predicted strength of the SVM classifier output. The m5C-miRNA signature performance was calculated using the R function “predict” to quantify the output intensity of the samples.

### Statistical analysis

The association between the output intensity of the m5C-miRNA signature and the expression of each individual miRNA was analyzed using Spearman’s correlation analysis. The statistical significance of the differences between two groups was evaluated by the Wilcoxon test. All statistical tests were performed using R version 4.1.2. All P values were two-sided, and *p* < 0.05 indicated statistical significance.

## Results

### Identification of the candidate m5C-miRNAs in serum

The workflow of this study was presented in **Supplementary Figure 1**. The extracted samples included 1507 GC, 260 GBM, 90 PAAD, 395 HCC, 49 BRCA, 442 BLCA, 90 SARC, 140 CRC, 1656 LC, 384 OV, 888 PRAD, and 706 ESCA samples together with 10295 normal samples. Next, all serum samples were randomly assigned to the training (3318 tumor samples and 5173 normal controls) or testing cohort (3289 tumor samples and 5122 normal controls). In the training cohort, subject age ranged 7–96 years (mean, 62.67 years), and 60.17% of the subjects were male. Participants in the validation cohort ranged in age from 7 to 100 years (mean, 62.47 years), and 59.28% of the subjects were male.

**Figure 1:**
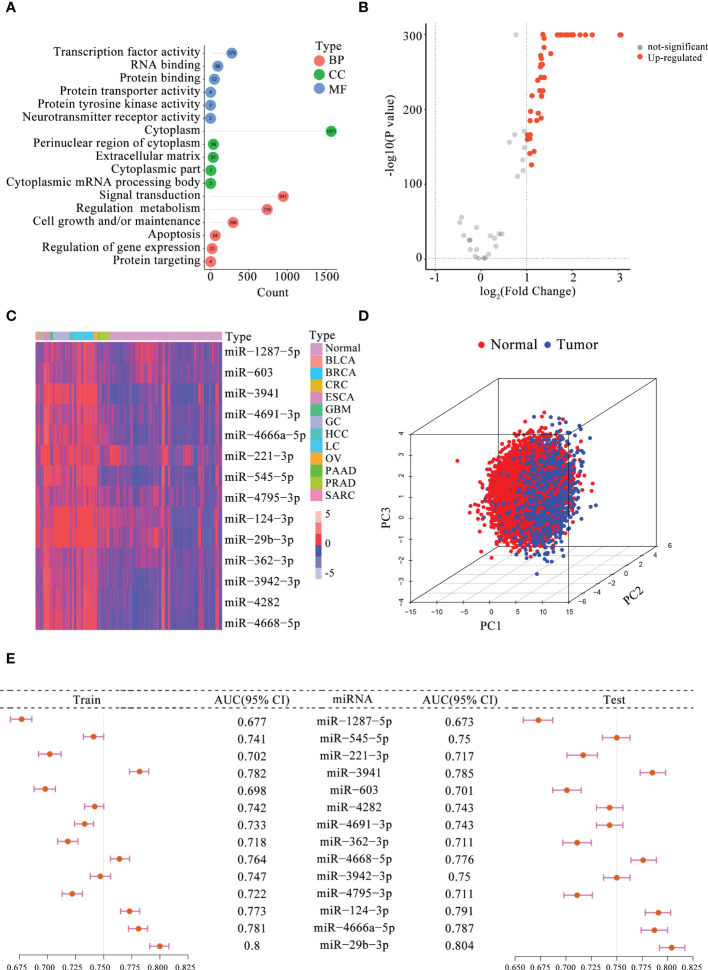
Determination of the candidate serum m5C-miRNAs. **(A)** GO analysis of 104 m5C-miRNAs. The number in the circle indicates the enriched gene count. BP, biological process; CC, cellular component; MF, molecular function. **(B)** Volcano plot presenting differentially expressed m5C-miRNAs between tumor and non-tumor samples (FDR < 0.05 and |fold change| > 1). **(C)** Expression heatmap of 14 candidate m5C-miRNAs in tumor and non-tumor samples. Red, upregulated miRNAs; blue, downregulated miRNAs. **(D)** Principal component analysis for 14 candidate m5C-miRNAs in tumor and non-tumor control. Red and blue dots represent tumor and non-tumor samples, respectively. **(E)** Diagnostic performance of each miRNA alone for identifying tumor samples in the training and testing cohorts. The AUC ranged from 0.673 to 0.804.

Based on previously published studies, 104 m5C-miRNAs from the 10 serum miRNA cohorts were selected for follow-up analyses. To explore the potential regulatory role of these m5C-miRNAs, we first performed GO enrichment analysis, and the results are presented in **Figure 1A**. These m5C-miRNAs were mainly enriched in signal transduction, regulation metabolism, cell growth and/or maintenance, apoptosis, and regulation of gene expression, and they were involved in RNA modification and tumor progression. Afterward, 43 m5C-miRNAs with differential expression between patients with tumors and normal controls in the training cohort were selected using the criteria of *p* < 0.05 and |fold change| > 1 (**Figure 1B**). Interesting, all of these m5C-miRNAs were upregulated in tumor samples. Finally, 14 candidate m5C-miRNAs were selected by LASSO regression analysis to construct the diagnostic signature. A remarkable separation between each tumor type and normal controls was observed on the basis of the unsupervised hierarchical clustering for 14 m5C-miRNAs (**Figure 1C**). Using principal component analysis for these miRNA profiles, we found that these miRNA signature displayed some capacity to differentiate between tumor and normal samples (**Figure 1D**). The results indicated that the identified miRNAs had distinct expression patterns between tumor and normal samples, facilitating the construction of the diagnostic signature. Then, the diagnostic capability of each candidate m5C-miRNA for tumor detection was evaluated. We discovered that the areas under the curve (AUCs) for individual m5C-miRNAs ranged from 0.677 to 0.800, revealing these miRNAs had certain ability to distinguish tumor samples from normal samples (**Figure 1E**). The diagnostic performance of the miRNAs was further confirmed in the testing cohort (**Figure 1E**). Collectively, these findings suggest that the 14 candidate m5C-miRNAs had promise as diagnostic biomarkers for tumor detection.

### Construction of the diagnostic model based on the candidate m5C-miRNAs

**Figure 2:**
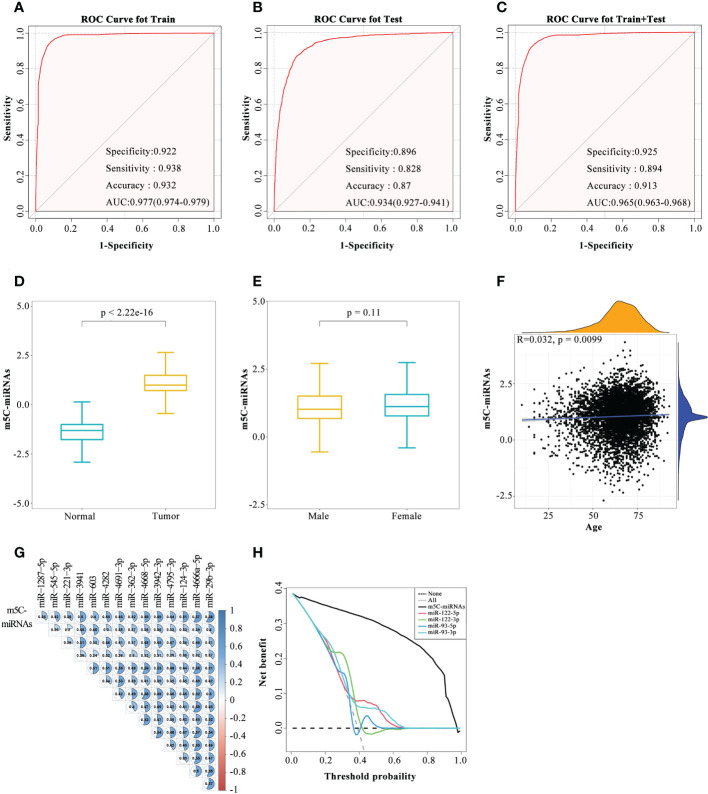
Development of the serum m5C-miRNA signature. **(A–C)** The diagnostic performance of the m5C-miRNA signature in discriminating tumor and normal samples in the training cohort **(A)**, testing cohort **(B)**, and combined training and testing cohort **(C)**. The AUC, specificity, sensitivity, and accuracy were calculated. **(D, E)** Differences in the output intensity of the m5C-miRNA signature between tumor and normal samples **(D)**, as well as between samples from male and female patients **(E)**. **(F)** Association between the output intensities of the m5C-miRNAs and the age of patients using Spearman’s correlation analysis. Yellow and blue represent the density of patients at different output intensities and different ages, respectively. **(G)** Association between the output intensities of the m5C-miRNA signature and 14 candidate m5C-miRNAs using Spearman’s correlation analysis. **(H)** Decision curve analysis revealed the difference of the net benefit between the m5C-miRNA signature and other serum miRNA biomarkers in the combined training and testing cohort.

According to the expression profiles of the 14 identified m5C-miRNAs, a diagnostic model (m5C-miRNA signature) was established for tumor detection by adopting the SVM method. As presented in **Figure 2A**, the ability of the m5C-miRNA signature to discriminate tumor and normal samples was significantly better than that each candidate miRNA alone in the training cohort [AUC = 0.977, 95% confidence interval (CI) = 0.974–0.979]. The diagnostic accuracy, specificity, and sensitivity were 93.2%, 92.2%, and 93.8%, respectively. High diagnostic performance was confirmed in the testing cohort, with accuracy of 87.0%, specificity of 89.6%, and sensitivity of 82.8%. The AUC of the diagnostic model in this cohort was 0.934 (95% CI = 0.927–0.941; **Figure 2B**). In the combined and testing cohort, the AUC (0.965), diagnostic accuracy (91.3%), specificity (92.5%), and sensitivity (89.4%) also revealed that the m5C-miRNA signature had satisfactory diagnostic utility (**Figure 2C**). We further validated the diagnostic ability of this model in an external validation cohort (AUC = 0.976, accuracy = 94.1%, specificity = 92.9%, sensitivity = 94.9%; **Supplementary Figure 2A**). The output intensity of the m5C-miRNA signature was remarkably higher in tumor groups than in the control group in the training cohort (**Figure 2D**). We also evaluated the output strength of the m5C-miRNA signature between different cancer types and observed the highest median value for the LC group (1.29278; **Supplementary Figure 2F**). Given that BRCA, OV, and PRAD were included in this work, we next examined the diagnostic power of the m5C-miRNA signature based on patient sex. No significant discrepancy regarding the output intensity of the signature was observed between male and female patients with tumors (**Figure 2E**). The m5C-miRNA signature also exhibited excellent diagnostic accuracy for both male and female patients (**Figures S2B, C**). We further performed correlation analysis to unravel the impact of age on the diagnostic performance of the m5C-miRNA signature. The results indicated no significant association of patient age with the m5C-miRNA output intensity (r = 0.032; **Figure 2F**). Therefore, the established m5C-miRNA signature appears to be a novel and independent biomarker for discriminating patients with cancer from healthy controls that is not affected by patient sex or age. Additionally, in both the training and testing cohorts, calibration curve analysis revealed that the predicted probability of cancer generated by the m5C-miRNA signature was nearly identical to the actual observed probability (**Figures S2D, E**). Using Pearson’s correlation analysis, the relationships of the m5C-miRNA signature with each candidate miRNA were assessed. Strikingly, there were significant positive associations between the output intensity of the signature and the expression of each candidate miRNA (**Figure 2G**). Moreover, decision curve analysis demonstrated that the m5C-miRNA signature had a net gain with absolute dominance over a wide range of decision threshold probabilities compared to previously reported miRNAs with great value in tumor diagnosis and prognosis (**Figures 2H**). Together, these results revealed that the m5C-miRNA signature constructed using 14 candidate miRNAs had excellent diagnostic performance for large-scale cancer detection.

### Evaluation of the diagnostic performance of the m5C-miRNA signature in different tumor types and clinical conditions

**Figure 3:**
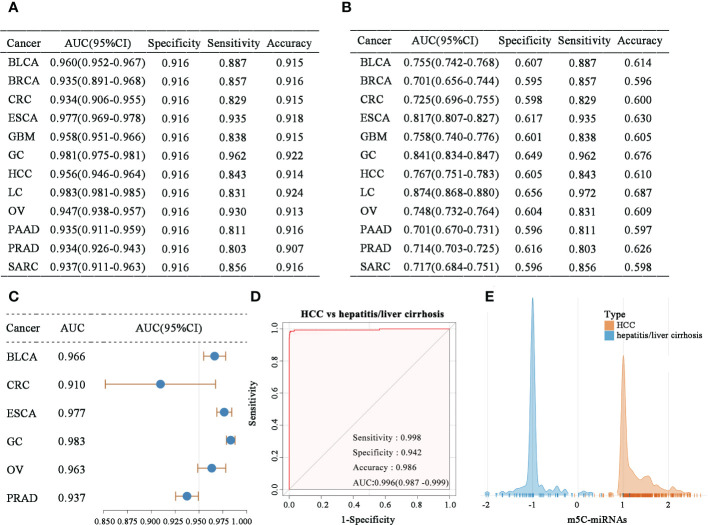
Evaluation of the diagnostic ability of the m5C-miRNA signature. **(A–B)** The diagnostic performance of the m5C-miRNA signature for discriminating each tumor type from non-tumor controls **(A)**, as well as each tumor type from all mixed samples of tumor and normal tissues **(B)**. **(C)** The early diagnosis capability of the m5C-miRNA signature in BLCA, CRC, ESCA, GC, OV, and PRAD. **(D)** The ability of the m5C-miRNA signature to discriminate patients with HCC from those with hepatitis/liver cirrhosis. **(E)** The sample densities of the HCC and hepatitis/liver cirrhosis groups at different m5C-miRNA output intensities.

In an analysis of samples of each tumor type combined with normal samples, the obtained m5C-miRNA signature displayed powerful ability to distinguish the cancer population from healthy people (**Figure 3A**). The m5C-miRNA signature also presented significantly high detection sensitivity when discriminating each tumor type from pooled serum samples of all tumor and normal controls (**Figure 3B**). This implies that the m5C-miRNA signature could accurately recognize the particular tumor type in more than 80% of patients. The m5C-miRNA signature also displayed satisfactory performance (AUC > 0.800) for differentiating LC, GC, and ESCA. Notably, the signature displayed excellent validity for the early diagnosis of some tumor types, including BLCA (AUC = 0.966), CRC (AUC = 0.910), ESCA (AUC = 0.977), GC (AUC = 0.983), OV (AUC = 0.963), and PRAD (AUC = 0.937; **Figure 3C**). Because hepatitis and liver cirrhosis are closely linked to the onset and progression of HCC and they frequently disturb the diagnosis of HCC, thereby delaying and treatment, the capability of the m5C-miRNA signature to differentiate hepatitis/liver cirrhosis samples from HCC samples was evaluated. As presented in **Figure 3D**, the diagnostic performance of the signature was not apparently affected by hepatitis or liver cirrhosis (HCC vs. hepatitis/liver cirrhosis: AUC = 0.996, specificity = 0.942, sensitivity = 0.998, accuracy = 0.986), which was further confirmed using the output intensities of the m5C-miRNA signature between patients with hepatitis/liver and those with HCC (**Figure 3E**). Cumulatively, these findings indicate that the m5C-miRNA signature has promise for distinguishing tumor types and detecting early-stage tumors, and its diagnostic performance was not affected by chronic diseases.

## Discussion

Despite exponential growth in our understanding of the pathogenesis of cancer and substantial investment in the development of effective treatments, cancer-specific mortality rates for most solid tumor types have barely changed for decades (22). At present, early cancer detection is the most effective and cost effective strategy for decreasing cancer-specific mortality (23, 24). However, clinically feasible non-invasive molecular markers for mass cancer screening are lacking. In this work, we developed an m5C-miRNA signature for large-scale cancer diagnosis based on the identified 228 target m5C-miRNAs and subsequently demonstrated its power for detecting tumors and discriminating tumor types.

Machine learning “learns” a model from past data in order to predict future data. A number of machine learning approaches such as the decision trees (DT), artificial neural networks (ANN), support vector machine (SVM), Naive Bayes (NB), and logistic regression (LR) have been developed to implement it (25). The current study revealed that SVM algorithm exhibited better performance in cancer genomic classification or subtyping than other algorithm (26). Thus, SVM algorithm was used in this study. Moreover, to mitigate the possibility of overfitting, we tuned the SVM parameters (C and gamma) in the case of nonlinear SVM. The parameters C (10^0^, 10^1^, and 10^2^) and gamma (10^1^, 10^0^, 10^-1^, 10^-2^, 10^-3^, 10^-4^, 10^-5^, and 10^-6^) in SVM were set, respectively. A total of 24 different parameter combinations were obtained. An optimal parameter combination (C: 10, and gamma: 0.1) was selected using 10-fold cross-validation.

Both genetic and epigenetic alterations are key factors that contribute to cancer progression, whereas aberrant epigenetic changes usually occur at the initial stage of tumor development (27, 28). m5C, as a common RNA modification type, has displayed significant value in the diagnosis of several cancers (9, 29). miRNAs have been identified as excellent candidate non-invasive biomarkers because of their high stability and abundance in serum (30, 31). Therefore, numerous studies have evaluated the diagnostic potential of various miRNAs, such as miR-320b, miR-125b, miR-221-3p, and miR-124-3p (32–35). However, the diagnostic performance of a single circulating miRNA is limited. The use of multiple miRNAs to construct an miRNA-based tumor diagnostic panel has outstanding advantages. Zekri et al. reported the high diagnostic accuracy (AUC = 1) of a three-miRNA panel consisting of miR-122, miR-885-5p and miR-29b combined with alpha-fetoprotein for the early detection of HCC (36). Zhang et al. established a two-miRNA panel (miR-185-5p and miR-362-5p) that displayed better potential than each miRNA alone in distinguishing patients with breast cancer from normal controls (37). In this study, we used 12 m5C-miRNAs to build a diagnostic model and discovered that this model possessed strong ability to discriminate tumor and non-tumor samples. The constructed m5C-miRNA signature exhibited excellent accuracy for large-scale cancer screening. The output intensity of m5C-miRNAs was positively associated with the expression of each candidate miRNA. Existing evidence suggests that these m5C-miRNAs, especially miR-221-3p, play key roles in tumorigenesis and tumor growth, metastasis, and prognosis. Zhou et al. found that miRNA-221-3p promoted the progression of head and neck squamous cell carcinoma and represent a non-invasive biomarker for diagnosis (38). miRNA-221-3p can serve as a biomarker for breast cancer prognosis (39). Kan et al. identified miRNA-221-3p as a serum marker of esophageal squamous cell carcinoma (40). Therefore, the m5C-miRNA signature constructed using these miRNAs had reliable diagnostic performance. Subsequent analysis revealed that the diagnostic performance of the m5C-miRNA signature in pan-cancer was unaffected by patients’ age and gender, and it had superior performance to previously reported miRNAs (e.g., miR-93, miR-122) (41, 42). Moreover, the m5C-miRNA signature displayed satisfactory specificity, sensitivity, and accuracy in distinguishing cancer types, especially LC, GC, and ESCA. Importantly, in the diagnosis of early-stage cancers, the m5C-miRNA signature had significantly better detection ability than traditional biomarkers, such as prostate-specific antigen, carcinoma antigen 125, carbohydrate antigen 72-4, carcinoembryonic antigen, and carbohydrate antigen 19-9 (43, 44).

This paper has some limitations that further experimental verification are needed to validate these findings. Although the m5C-miRNA signature had promising performance for the early diagnosis of six tumor types, this study was limited by the lack of corresponding staging information for other cancers, which prevented us from evaluating the diagnostic utility of the signature for these cancers in the early stage. Accordingly, the performance of the m5C-miRNA signature in the diagnosis of other early-stage cancers remains to be further assessed.

## Conclusions

This study is the first to establish a novel m5C-miRNA signature with high accuracy and sensitivity for pan-cancer diagnosis and cancer type discrimination and to unveil the value of serum m5C-miRNAs in tumor detection. Furthermore, the diagnostic ability of the developed m5C-miRNA signature in pan-cancer was not affected by patients’ age or gender or by the presence of noncancerous diseases. More importantly, the m5C-miRNA signature displayed superior sensitivity in early-stage tumor diagnosis. To conclude, the m5C-miRNA signature highlights the feasibility of identifying non-invasive and cost effective biomarkers for mass cancer screening.

## Data availability statement

The original contributions presented in the study are included in the article/**Supplementary Material**. Further inquiries can be directed to the corresponding authors.

## Ethics statement

Ethical review and approval was not required for the study on human participants in accordance with the local legislation and institutional requirements. Written informed consent for participation was not required for this study in accordance with the national legislation and the institutional requirements.

## Author contributions

Conceptualization: FT, YL, JZ, YO, and SZ. Methodology: FT, SZ, YS, and YX. Investigation: YG, and SZ. Visualization: YL, YG, YS, and YO. Funding acquisition: FT, YO, and SZ. Project administration: JZ, and SZ. Writing original draft: FT, YL, JZ, YO, and SZ. All authors contributed to the article and approved the submitted version.

## Supplementary material

Supplementary Figure 1 Overview of study design.

Supplementary Figure 2 Analysis of the diagnostic ability of the m5C-miRNA signature. **(A–C)** The diagnostic performance of the m5C-miRNA signature in discriminating tumor and normal samples in the external validation cohort **(A)**, female populations **(B)**, and male populations **(C). (D–E)** The diagnostic performance of the m5C-miRNA signature vs observed cancer in the training cohort **(D)**, and validation cohort **(E)**. **(F)** Differences in the output densities of m5C-miRNAs signature between tumor and non-tumor control samples.
