# Supplementary figures and images for: Construction of a serum diagnostic signature based on m5C-related miRNAs for cancer detection
Source: Front Endocrinol (Lausanne). 2023 Jan 27;14:1099703. doi: 10.3389/fendo.2023.1099703 (PMC9911864; doi:10.3389/fendo.2023.1099703)

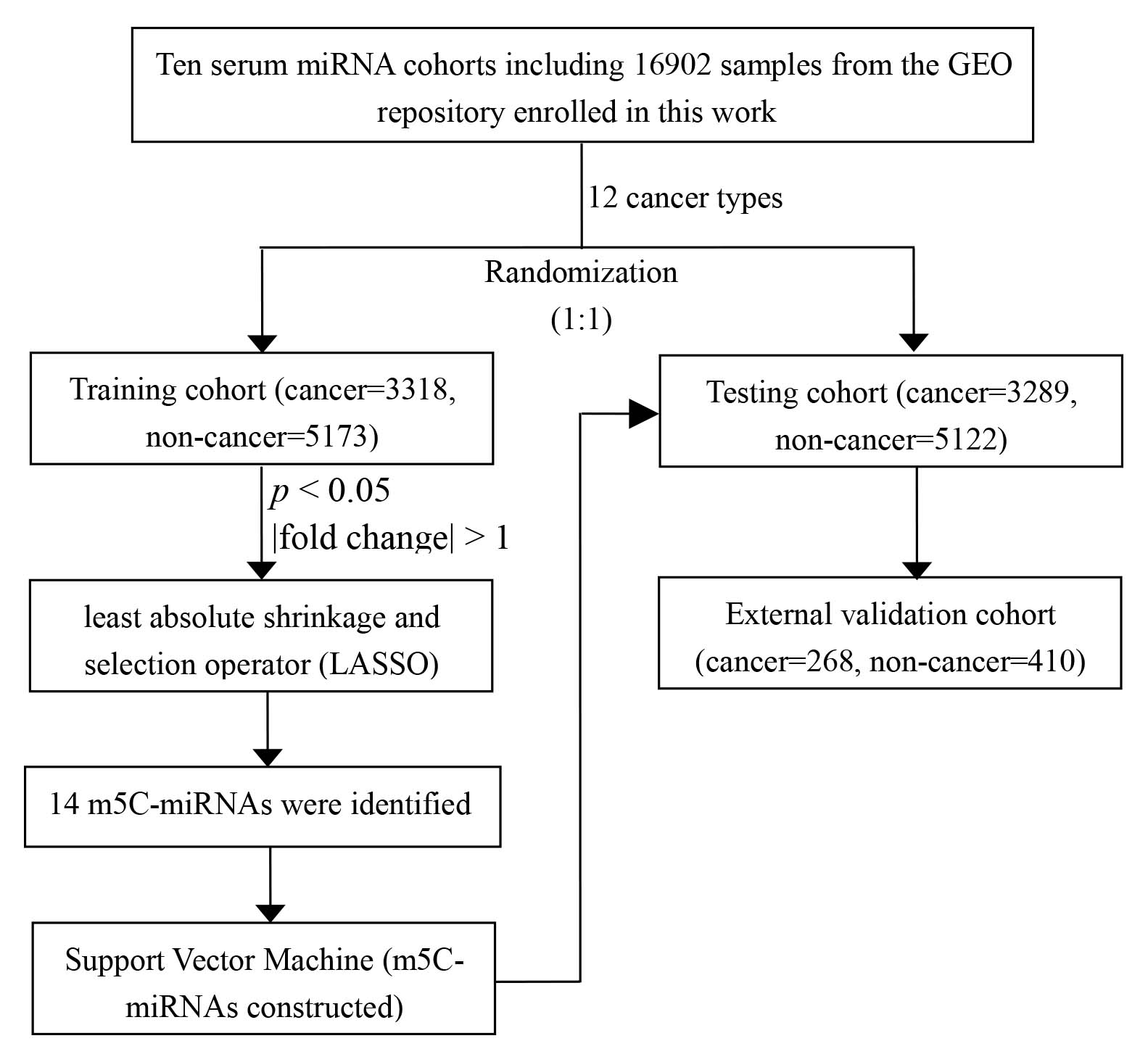

Supplement: Supplementary Figure 1 — Overview of study design. [file Image_1.jpeg]

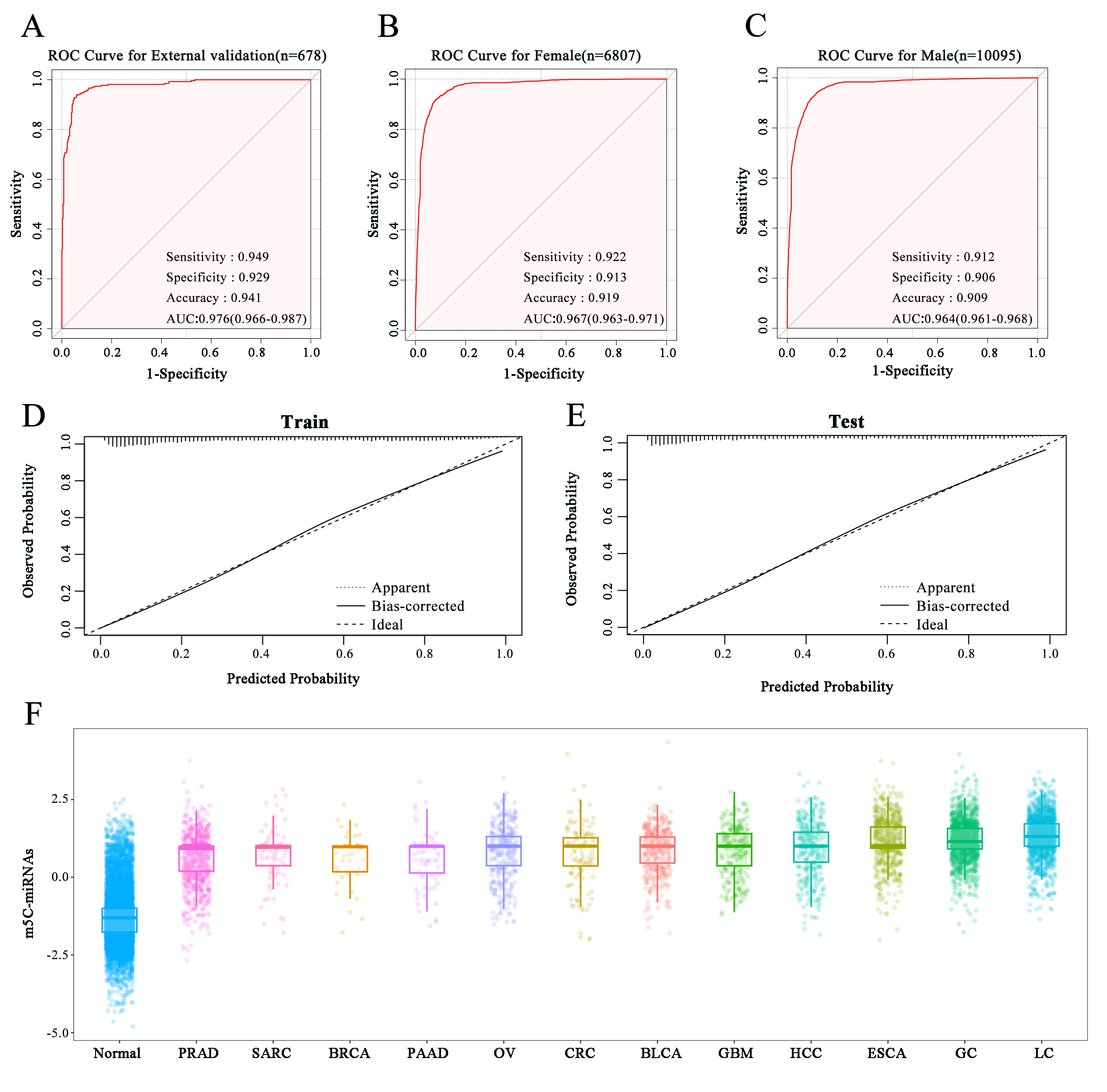

Supplement: Supplementary Figure 2 — Analysis of the diagnostic ability of the m5C-miRNA signature. (A–C) The diagnostic performance of the m5C-miRNA signature in discriminating tumor and normal samples in the external validation cohort (A), female populations (B), and male populations (C). (D–E) The diagnostic performance of the m5C-miRNA signature vs observed cancer in the training cohort (D), and validation cohort (E). (F) Differences in the output densities of m5C-miRNAs signature between tumor and non-tumor control samples. [file Image_2.jpeg]
